# Memristive Behavior in Carrier Accumulation-Based Optical Modulators

**DOI:** 10.1021/acs.nanolett.5c03443

**Published:** 2025-09-29

**Authors:** Alexander Korneluk, Katarzyna Brańko, Tomasz Stefaniuk

**Affiliations:** Faculty of Physics, 49605 University of Warsaw, Pasteura 5 St., 02093, Warsaw, Poland

**Keywords:** electro-optical modulators, field effect modulation, electro-optical memristors, indium−tin-oxide, ionic transport, electrochemical metallization, index modulation

## Abstract

Memristive switching and field-effect modulation form the basis of many optoelectronic devices, yet despite their complementary properties, they are typically realized in separate architectures. Here, we demonstrate an optoelectronic platform that combines carrier accumulation/depletion (CAL/CDL) and electrochemical metallization (ECM) effects within a single device. By engineering a Ag/ITO/SiO_2_/Ag stack and tuning the ITO carrier concentration, we achieve electrically driven transitions between volatile and nonvolatile optical states. Spectroscopic ellipsometry and electrical measurements, enhanced by well-defined optical resonances and a large active area, reveal that low-voltage modulation originates from field-induced carrier redistribution at the ITO/SiO_2_ interface (CAL/CDL), while long-term optical drift and current evolution are attributed to ECM-mediated silver ion migration and filament formation. The coexistence and controllable interplay of both effects provide a pathway toward multifunctional optoelectronic components capable of operating across distinct memory and modulation modes, with implications for neuromorphic computing and hybrid photonic in-memory computing technologies.

## Graphical abstract

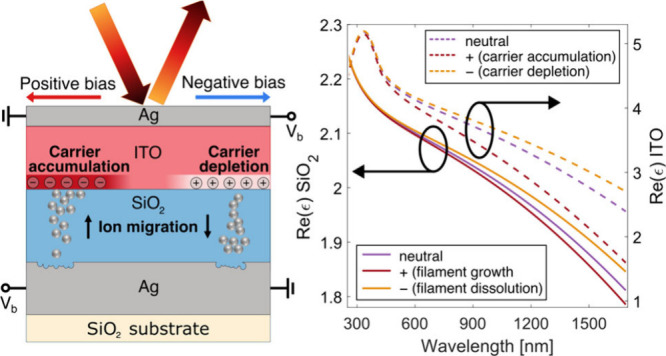

Recent advances in nanostructuring have driven rapid progress in photonics technologies, enabling more efficient devices with functionalities that go far beyond those of conventional optics. As the field evolves, growing attention is directed toward the real-time tuning of electromagnetic responses. From an energy efficiency and speed perspective, a highly promising approach emerges from the family of electrically induced modulation techniques that exploit the physics of the semiconductor–oxide interface.^1^ Specifically, the mentioned strategy relies on the formation of a high- or low-density free carrier layer in proximity to the interface induced by an applied voltage. Unlike traditional bulk carrier modulation methods, which require large carrier injection and suffer from high energy dissipation,^2^ a carrier accumulation (CAL) or depletion (CDL) layer effect confines charge carriers within an ultrathin interfacial region, leading to unity order local change of the refractive index, a phenomenon first observed in a Au/ITO/SiO_2_/Au stack.^3^ Typical CAL/CDL structures employ transparent conductive oxides (e.g., ITO, AZO) as semiconductors paired with electrically insulating dielectrics (SiO_2_, Al_2_O_3_, HfO_2_, Si_3_N_4_) and metal electrodes (Au, Ag), creating tunable carrier density profiles at nanoscale interfaces.^4–12^ Unfortunately, since the effect occurs in a very small volume and the accumulation layer thickness (a few nanometers or less) is much smaller than the optical mode cross-section, the overall change in the structures’ optical response is minimal. To address this challenge, integrating waveguide geometry has been used as an effective strategy, extending the interaction length and substantially enhancing light modulation performance.^13–15^

Structures with designs nearly identical with the described modulators—fabricated from the same or similar materials—are also employed in a distinct research domain: memristive optoelectronics. These nonvolatile switching devices modulate electrical resistance^16,17^ or, as recently demonstrated, also optical response,^18–20^ based on the history of the applied voltage or current and are actively explored for applications in neuromorphic computing, memory storage, and in-memory computing.^21^ Among various memristive switching mechanisms, particular attention should be given to those relying on filamentary conductive pathways such as electrochemical metallization (ECM) devices. In ECM systems, voltage application induces metal atoms from an active electrode (such as Ag, Cu, or Ni) to ionize, migrate through an insulating layer (e.g., SiO_2_, Al_2_O_3_), and form a conductive filament, enabling a low-resistance state. This process is reversible, as the filament dissolves during the reset operation, restoring the high-resistance state.^22^ The ECM mechanism in memristors offers several advantages, including low energy consumption, fast switching speeds, and high endurance, making it a promising approach for nonvolatile memory applications.^21,23^ However, understanding the dynamics of nanoscale filament formation and dissolution remains difficult due to their extremely small dimensions. Due to the limited availability of reliable techniques, investigations are essentially restricted to transmission electron microscopy or conductive atomic force microscopy, both necessitating invasive cross-sectioning of the sample.^24–29^ In this context, the combined use of spectroscopic ellipsometry and electrical measurements is particularly valuable for in operando investigations of both the physical evolution and the underlying mechanisms governing filament behavior.^18^

Interestingly, despite the use of similar materials and geometries in devices utilizing the CAL/CDL effect of ECM-based systems, no reports in the literature indicate that both effects occur simultaneously. This absence suggests a clear separation between these two research domains. Yet, understanding and distinguishing the interplay between these mechanisms is crucial, as each fundamentally impacts a device’s optical and electrical properties, with direct consequences for modulation dynamics, switching behavior, and long-term reliability. On the other hand, harnessing both effects in a unified platform not only enables precise control over volatile and nonvolatile functionalities but also fully exploits their complementary nature. The CAL effect, with its purely electronic origin, offers fast response, inherent reversibility, and freedom from the stochastic variability of ionic motion and filamentary growth, making it ideally suited for short-term volatile functionalities reminiscent of synaptic plasticity in biology. In contrast, ion-driven ECM processes naturally yield long-term nonvolatile states that provide robust memory retention. Their coexistence within a single device paves the way for multifunctional optoelectronic systems such as neuromorphic architectures, nonvolatile memories, and hybrid photonic in-memory computing technologies.

In this Letter, we report the first experimental demonstration of the coexistence and tunable interplay between CAL and ECM effects within a single ITO-based optoelectronic platform. By precisely engineering the carrier concentration in the ITO layer and incorporating a Ag reservoir electrode, we enable distinct transitions between volatile and nonvolatile optical switching regimes under low-power operation. The device architecture supports selective activation of each mechanism through controlled adjustment of the bias amplitude and duration, thereby addressing the previously unachieved integration of both effects in a single system.

The investigated structure was fabricated using an electron beam evaporator (Lesker PVD75) with precision masking, as illustrated in Figure 1a (see also the methods section in the Supporting Information). It features silver electrodes with thicknesses of 100 nm (bottom) and 20 nm (top). To mitigate silver’s tendency to agglomerate, a 1 nm germanium wetting layer was introduced to improve film morphology.^30^ Silver was chosen for its high reflectivity and low optical losses in the visible and NIR, which are essential for forming a high-quality optical cavity and enhancing signal extraction from the active region. On the other hand, silver is widely utilized in ECM memristors due to its high ionic mobility and excellent electrical conductivity, which promote rapid switching and efficient formation of conductive filaments.^16,31^

**Figure 1:**
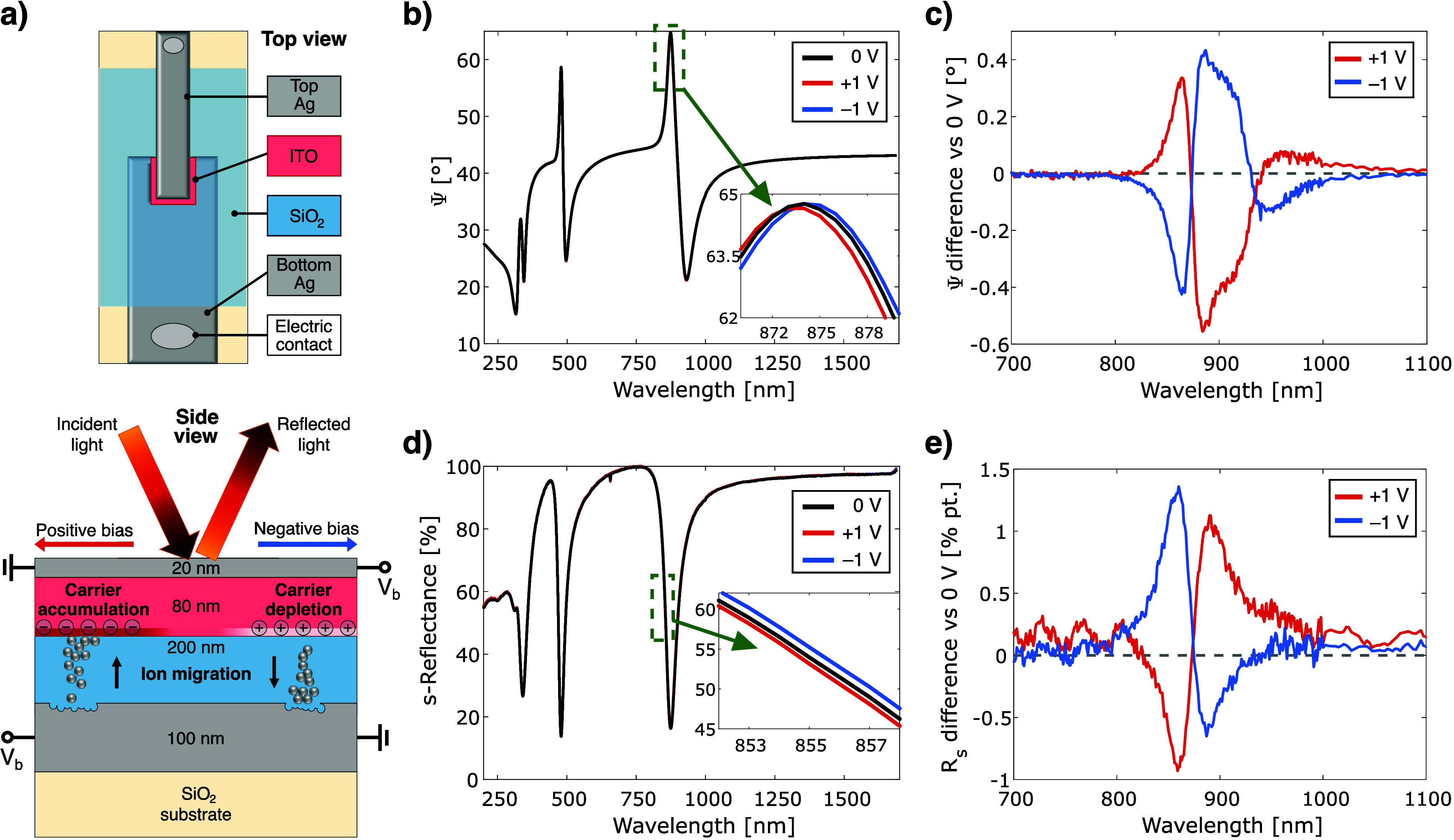
(a) A conceptual diagram demonstrating the device’s geometry, operating principles, and the methodology of optical measurement. (b) The ellipsometric parameter Ψ, measured at an incidence angle of 65°, obtained under different bias conditions. (c) Spectral variation in the Ψ function for positive and negative bias voltages. (d) s-Reflectance, measured at an incidence angle of 65°, obtained under different bias conditions. (e) Spectral variation in the s-reflectance for positive and negative bias voltages. The insets provide a close-up of one of the optical resonances.

Between the electrodes, there is a 100 nm-thick ITO layer, which typically serves as the active layer in carrier accumulation-based modulators, as well as a 180 nm-thick SiO_2_ layer that is used in both modulators and memristors, serving as an insulator in the former and as a switching layer in the latter. By optimizing the ITO deposition process through oxygen plasma treatment,^32,33^ an ITO layer with a concentration of *N* = 1.51 × 10^20^ cm^–3^ was successfully obtained without requiring an annealing step. Literature studies indicate that this concentration should ensure high efficiency in the formation of accumulation (or depletion) layers at the semiconductor–dielectric interface.^34^ Regarding the selection of SiO_2_, it appears to be a suitable candidate for the experiment, as it is widely utilized as a switching layer in ECM memristors due to its CMOS compatibility, scalability, and ability to support controlled filament formation, particularly in combination with silver electrodes.^19,21,24,35,36^ With its large 2.5 mm × 2.5 mm active area, the device stands out from standard designs, offering enhanced sensitivity in electrical and optical readouts and enabling detection of subtle effects. Each sample contains multiple pixels, allowing different testing procedures while maintaining nearly identical material and geometric parameters due to shared fabrication.

The sample’s optical response under electrical stimulation was measured using an RC2 ellipsometer (J.A. Woollam Co.) with dual-rotating compensators placed before and after the sample. The optical spot defined by the focusing probes had a 400 μm diameter and covered an area much smaller than that of a single pixel. Spectroscopic ellipsometry (Ψ and Δ) and polarization-dependent reflectance data were collected at a 65° incidence angle, selected to induce strong optical resonances and approach the Brewster angle, maximizing ellipsometric sensitivity. Ψ represents the amplitude ratio change between p- and s-polarized light, while Δ is the corresponding phase shift. Together, they offer a detailed view of the polarization changes.

Figure 1b shows the Ψ function measured for the sample under investigation, revealing multiple maxima corresponding to optical Fabry–Pérot resonances within the structure. Upon application of an external voltage, these resonance peaks shift either to the left or right depending on the polarity of the applied bias (see inset). The most pronounced changes in the Ψ curve are observed in spectral regions with the steepest slopes (Figure 1c), where the sensitivity to optical modulation is highest. Similar behavior has been reported for related structures and is commonly attributed to the formation of accumulation or depletion layers at the oxide–semiconductor interface under positive or negative voltage, respectively.^11,13,14^ A clear modulation is also observed in the Δ function (Figure S1) as well as in the reflectance under both s-polarized (Figures 1d and 1e) and p-polarized (Figure S2) illumination. In the following analysis, we will focus on the Ψ curve, as it offers more reliable and precise information about thin-film characteristics, benefiting from the inherent strengths of ellipsometry.

The unique design of our samples, characterized by a large active area and high-quality optical resonances, enables the observation of additional phenomena beyond first-order responses arising from multiple modulation cycles. Figure 2a presents the Ψ function values at a wavelength of 890 nm under varying electrical stimulations (constant voltage kept for 13 s) for a pristine pixel with no prior bias application. The blue, red, and black dots correspond to negative, positive, and zero applied voltage. Despite the thick dielectric layer, the system exhibits clear optical modulation at voltages as low as 0.5 V, with the amplitude increasing systematically with voltage. Notably, the system exhibits a gradual drift toward lower Ψ function values, behavior that cannot be solely attributable to carrier accumulation or depletion effects. Doubling the voltage does not significantly accelerate this drift. The steady-state current responses under different voltage levels depicted in Figure 2b provide further insights into the system’s state. For each cycle, the leakage current remains at approximately 0.15 μA, which, considering the large active area of the structure, corresponds to a current density of 2 × 10^–14^ A/μm^2^, indicating an almost perfect suppression of current flow. This confirms that the applied electrical stimulation throughout the entire sequence remained sufficiently low to prevent short-circuiting and preserve the CAL/CDL effects. Interestingly, a slight and consistent increase in current is observed under a +1 V bias, whereas it completely vanishes upon each application of a negative voltage. The origin of this behavior will be discussed in detail in a later section of this Letter.

**Figure 2:**
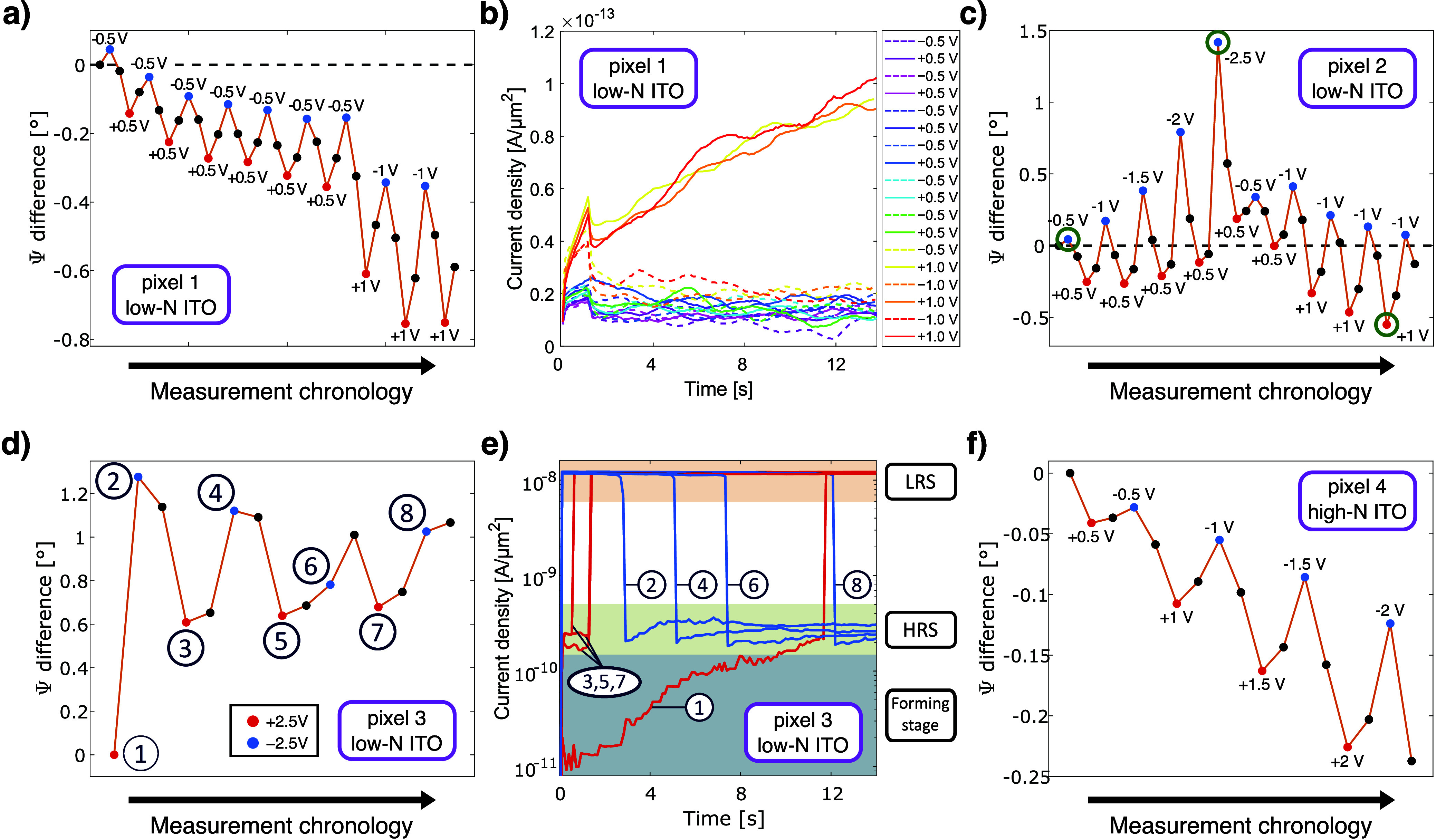
(a) Modulation of the Ψ function (with respect to the initial value of Ψ) under applied constant voltage for pixel #1 at λ = 890 nm and (b) the corresponding current flow through the structure. (c) Modulation of the Ψ function for pixel #2 at different testing procedures. The corresponding current flow can be found in the Supporting Information in Figure S3a. The green circles indicate measurement data used in the development of an ellipsometric model to determine the permittivity of the SiO_2_ and the interfacial layer between SiO_2_/ITO (see Figure 3). (d) High-voltage modulation of the Ψ function for pixel #3 at λ = 890 nm and (e) the evolution of the corresponding current curves over time, measured during subsequent cycles. (f) Modulation of the Ψ function under applied voltage for a sample (pixel #4) with a high carrier concentration at λ = 1035 nm.

In the next phase of the study, we examined whether the application of asymmetrical voltage values could mitigate the observed drift or alter its direction, as illustrated in Figure 2c. A fresh pixel was employed to eliminate any potential influence of prior measurements on the results, following the same approach as that in the previous step. The data indicate that the initially observed trend was successfully reversed, gradually shifting toward higher Ψ function values. However, once symmetrical voltages were reapplied at the midpoint of the procedure (after the −2.5 V point), the original tendency for downward drift was reinstated and the Ψ function returned to its initial value. This finding indicates that modulation of the optical signal around a predefined set point is achievable in the examined structure but requires asymmetrical voltage stimulation. We also observed that the applied test procedure led to a further increase in the leakage current, reaching a density of 10^–12^ A/μm^2^ (see Figure S3a in the Supporting Information), which is still a few orders of magnitude smaller than reported for other CAL/CDL systems in the literature.^16,17,19^ While the resulting current density still remains almost negligible, it is worth noting that the current increases were again observed predominantly under positive voltage bias. Motivated by this recurring behavior, we proceeded to investigate the underlying mechanism of the leakage current increase by subjecting a new, pristine pixel to a higher voltage stimulation. It is also worth emphasizing that the described trends—particularly in the optical domain—were remarkably reproducible across different pixels as well as across distinct samples, provided that the same testing procedure and voltage sequence were applied (for example, see the results for pixel 6 in Figures S3b, measured using the same procedure as pixel 2).

Figure 2d and 2e present the ellipsometric data along with the corresponding time-dependent current data, obtained under elevated voltage conditions. In contrast to previous measurement cycles, the optical modulation of the structure noticeably weakens with successive switching events. However, the most significant difference compared to earlier tests lies in the shape and magnitude of the current curves. During the first cycle (point #1), the +2.5 V signal (represented by the curve starting at 10^–11^ A/μm^2^) gradually increases to approximately 2 × 10^–10^ A/μm^2^. Just before the 12th second of measurement, a sudden current surge occurs, reaching around 10^–8^ A/μm^2^. Since the current magnitude changes by nearly 2 orders of magnitude, we define 2 × 10^–10^ A/μm^2^ as the high-resistance state (HRS) and 10^–8^ A/μm^2^ as the low-resistance state (LRS) for further analysis. In the second part of the first cycle (point #2), the system initially remains in the LRS state before switching to the HRS state just before the third second. This trend repeats in subsequent cycles, where under positive voltage, the system transitions to the LRS state, while under negative voltage, it switches back to the HRS state. However, in each successive cycle, while the switching event itself is almost instantaneous, its onset shifts progressively later in the bias sequence, as will be explained in the following discussion of the mechanisms involved.

Finally, we investigated the effect of increasing the carrier concentration in the ITO layer on the system’s performance, as this parameter is expected to significantly affect the strength of the CAL/CDL effect by eliminating internal electrostatic potential gradients, thereby suppressing the formation of charge accumulation/depletion layers.^34,37^ By fine-tuning the oxygen plasma parameters during the deposition, we successfully fabricated a structure with an ITO layer exhibiting a carrier concentration of *N* = 7.75 × 10^20^ cm^–3^ while keeping all other structural parameters unchanged. The increase in carrier concentration modified the optical properties of the ITO layer, leading to a spectral shift and reshaping of the resonance features (Figure S3d in the SI). However, apart from a noticeable reduction in Ψ modulation strength under varying applied voltages (Figure 2f), no other significant impact on the structure’s performance was observed. The previously described drift in the optical properties of the system toward lower Ψ values still persists when symmetric voltages are applied.

All of the observed and described dependencies can be attributed to effects that have been independently reported earlier for modulator devices based on carrier CAL/CDL effects and for memristive structures. The primary mechanism responsible for the switching behavior of the proposed structure is likely the effect of carrier accumulation and depletion. Although the applied SiO_2_ layer is relatively thick, which hinders the occurrence of this phenomenon, the combination of a well-defined optical resonance, a large active area, and highly sensitive ellipsometric measurements enabled its detection even at low applied voltages. Additional supporting arguments include the absence of current flow in the system and the observed reduction in modulation strength when ITO is used with a high carrier concentration. This reduction occurs because the high initial free carrier density effectively screens the applied electric field, thereby limiting the ability to induce additional charge accumulation at the interface.

On the other hand, the observed optical drift and gradual increase in leakage current can be attributed to the ECM effect. Voltage application induces oxidation at the electrode interface followed by silver ion migration into the insulating SiO_2_ layer. This process is inherently asymmetric due to the polarity-dependent nature of ion transport and electrochemical reactions. Under positive bias, oxidation occurs at the bottom, thick electrode, which acts as a reservoir of mobile Ag^+^ ions, enabling continuous filament nucleation and growth. The efficiency of this process is sufficiently high that upon voltage removal, the system does not revert to its initial optical state but instead exhibits a persistent shift toward lower Ψ values. Due to this favored filament growth under positive bias, the current increase observed in Figures 2b and 2e takes place under positive rather than negative bias. At the same time, if the applied voltage is too high, then the metallic filament becomes increasingly stabilized and partially preserved from the previous cycle. Its rupture then requires a longer time under bias to fully dissolve the residual conductive pathway, resulting in a delayed reset process compared to the initial cycles, as illustrated in Figure 2e.

In contrast, the top electrode is partially ionically insulated from the dielectric by a 100 nm thick ITO layer, whose dense lattice and reduced potential drop (with respect to SiO_2_) collectively suppress ion mobility and injection.^38^ Moreover, due to its reduced thickness, the top electrode provides fewer mobile Ag^+^ ions, which directly affects the kinetics of filament formation and dissolution.^18^ As a result, under negative voltage bias, ion extraction from the SiO_2_ switching layer dominates, suppressing the formation and stabilization of conductive filaments. This process can be balanced by applying asymmetric voltages, as illustrated in Figure 2d.

The current trends obtained under strong voltage stimulation at ±2.5 V indicate that filament formation is completed under positive voltages, transitioning the system to the LRS, whereas its dissolution and rupture occur under negative voltages, leading to the HRS. This results in the characteristic shape of a bipolar hysteresis loop, typical of an ECM-type memristor (Figure S4a). The gradual reduction in modulation strength under these conditions can be explained by the increasingly frequent short-circuiting of the system, which ultimately limits the carrier CAL/CDL effect.

SEM images of pixels 1 and 3 further substantiate the proposed ionic dynamics (Figure S5 and SI). At low bias on pixel 1, silver nanoclusters migrate within the SiO_2_ layer, whereas at higher bias on pixel 3, fully developed dendrites are observed. Their nucleation at the Ag/ITO interface indicates a bootstrapping mode, in which nanoclusters act as bipolar nanoelectrodes, undergoing upstream oxidation and downstream reduction to relay material toward the counter electrode.^24^ Consequently, filament growth proceeds stepwise rather than by continuous reduction at the ITO/SiO_2_ interface. Even after repeated switching under opposite polarities, only a few nanoclusters appear in the ITO region, underscoring its role as an effective ionic barrier.

While ECM effects strongly influence the system’s response, they alone cannot explain all experimental findings. Considering a sample with a high carrier concentration in the ITO layer, if the modulation mechanism were solely ECM-based, an increase in carrier concentration should have enhanced modulation strength. A higher carrier concentration would be expected to make the ITO layer an even more effective barrier for ions from the top electrode, facilitating efficient silver ion removal from the switching layer for a negative voltage. Simultaneously, improved electronic conductivity promoted faster oxidation and ion release from the bottom electrode. However, none of these effects were observed, further indicating the coexistence of both ECM effects and carrier accumulation in the studied device (see also extended discussion in the SI).

So far, our analysis has focused on data obtained from direct optical measurements. However, based on ellipsometric data (see the points marked with green circles in Figure 2c), a dielectric function model can be constructed to determine the permittivity ε of individual layers. We developed such a model, calibrated it with a pristine sample, and used it to analyze the permittivity (ε) of the SiO_2_ film and the interfacial ITO layer near the SiO_2_/ITO interface under an applied voltage, as shown in Figure 3.

**Figure 3:**
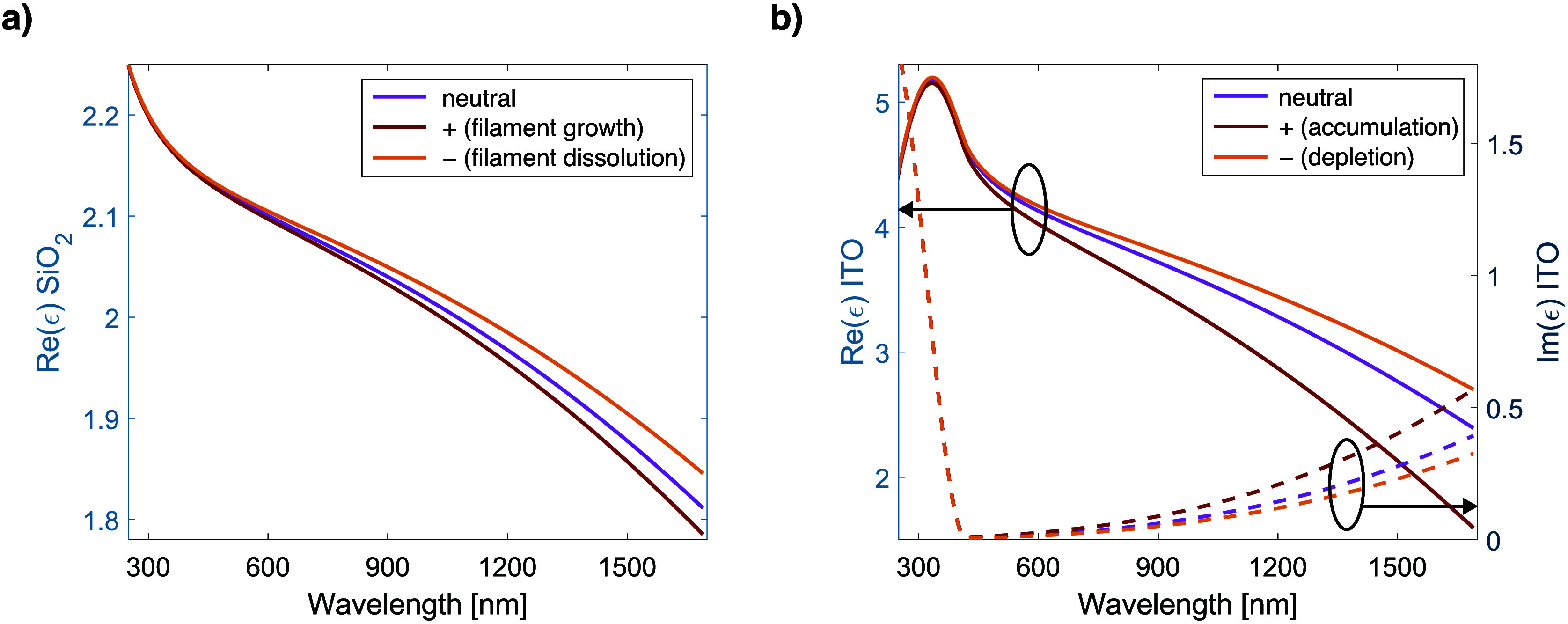
Permittivity values for the (a) SiO_2_ and (b) interfacial low-*N* ITO layer (pixel 2), extracted from ellipsometric data and modeling.

According to the developed model, the initial ε­(SiO_2_) deviates from bulk values, especially in the near-infrared, where its curvature shifts to a Drude-like profile—likely due to spontaneous Ag cluster migration from the bottom electrode during or after e-beam deposition.^18,39^ Applying a negative voltage affects the ε­(SiO_2_) curve by decreasing the density of silver nanoclusters and enhancing the insulating properties of the SiO_2_ layer. Simultaneously, the negative voltage depletes charge carriers at the SiO_2_/ITO interface, reducing their concentration and shifting the real part of the ε­(ITO) curve upward in the long-wavelength region. Conversely, applying a positive voltage reverses these trends: charge carriers accumulate at the interface, and silver nanoclusters redeposit in the SiO_2_ layer, both effects causing a downward shift of ε curves at longer wavelengths and increasing the imaginary part of ε­(ITO).

Finally, to demonstrate how CAL and ECM mechanisms emulate short- and long-term synaptic plasticity, in Figure 4 we show time-dependent measurements for pixel #5 with low-*N* ITO. Under asymmetric voltages (−0.7 V/+1 V), stable and highly repeatable optical cycles are obtained (Figure 4a) with low currents (<3 × 10^–11^ A/μm^2^, Figures 4b and S4b), confirming controlled metal cluster movements and/or dendrite formation/dissolution. The applied bias induces both volatile (CAL/CDL) and nonvolatile (ECM) responses, evident in the Ψ modulation cycles (Figure 4c), with levels preserved and further stabilized over time (Figure 4d). Multilevel nonvolatile states are also demonstrated (Figure 4e), where negative-bias pulses shift Ψ downward and positive bias enables higher levels, all clearly resolved optically.

**Figure 4:**
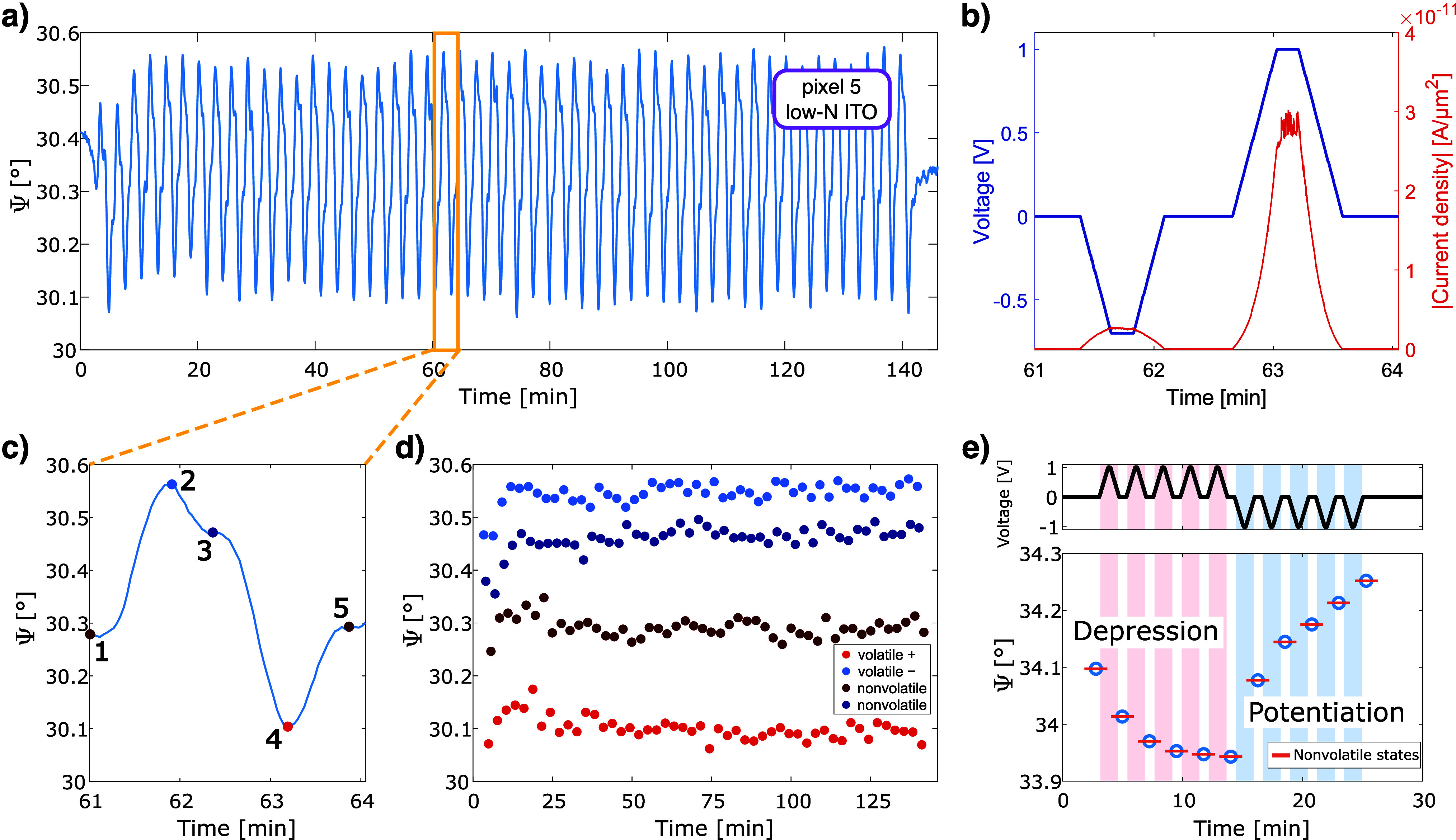
(a) Modulation of the Ψ function under an applied asymmetric voltage of −0.7 V/+1 V for pixel #5 at λ = 880 nm. (b) A single modulation cycle with the corresponding measured current flow through the structure. (c) Close-up of the Ψ function during one cycle, with labeled points corresponding to different device states: nonvolatile (0 V), volatile (−0.7 V), nonvolatile (0 V), volatile (+1 V), nonvolatile (0 V). (d) Averaged Ψ values of the volatile and nonvolatile states over multiple cycles, with color coding consistent with the labels in panel (c). (e) Illustration of the multilevel analogue operation of the device, with low-*N* ITO, demonstrating access to a range of distinct nonvolatile optical states. The shaded areas indicate the time intervals when voltages of +1 V or −1 V were applied.

In summary, we demonstrate a metal-oxide–semiconductor device that uniquely combines an electronic mechanism (carrier accumulation/depletion, CAL/CDL) with an ionic mechanism (electrochemical metallization, ECM) within a single platform. CAL/CDL enables fast, volatile optical modulation, while ECM introduces stable, nonvolatile states through silver ion migration. The implementation of a robust optical readout, performed in parallel with electrical excitation, allows intermediate and multilevel states to be resolved with high fidelity and renders the device operation largely insensitive to stochastic filament rupture. Our findings may help bridge the gap between photonic modulators and memristive systems, where such hybrid behavior could not only improve the emulation of biological synapses but also enhance the versatility of memristive devices for neuromorphic applications, potentially enabling real-time adaptation, multilevel weight tuning, and efficient in-memory computing architectures.

## Supplementary Material



## Notes

Delta and *R*_p_ spectra and the corresponding changes in these functions under applied voltage, current evolution curves for pixel #2, repeatability of the asymmetric voltage stimulation performed on a different (#5) pixel and the corresponding current flow through the structure, Ψ spectrum and Ψ modulation over wavelength for pixel #4 with high carrier density ITO and the corresponding current evolution curves, current–voltage characteristics of pixel 3 and pixel 5, SEM images of cross sections of pixels 1 and 3, additional result discussion and methods’ description (PDF)
