# Supplementary material for: Memristive Behavior in Carrier Accumulation-Based Optical Modulators
Source: Nano Lett. 2025 Sep 29;25(40):14600–7. doi: 10.1021/acs.nanolett.5c03443 (PMC12512192; doi:10.1021/acs.nanolett.5c03443)
Supplement: Supplementary file 1 [file nl5c03443_si_001.pdf]

Supporting Information – Memristive behavior in carrier-accumulation based optical modulators

*Alexander Korneluk, Katarzyna Brańko, Tomasz Stefaniuk**

University of Warsaw, Faculty of Physics, Pasteura 5 St., 02093, Warsaw, Poland

Additional Experimental Results

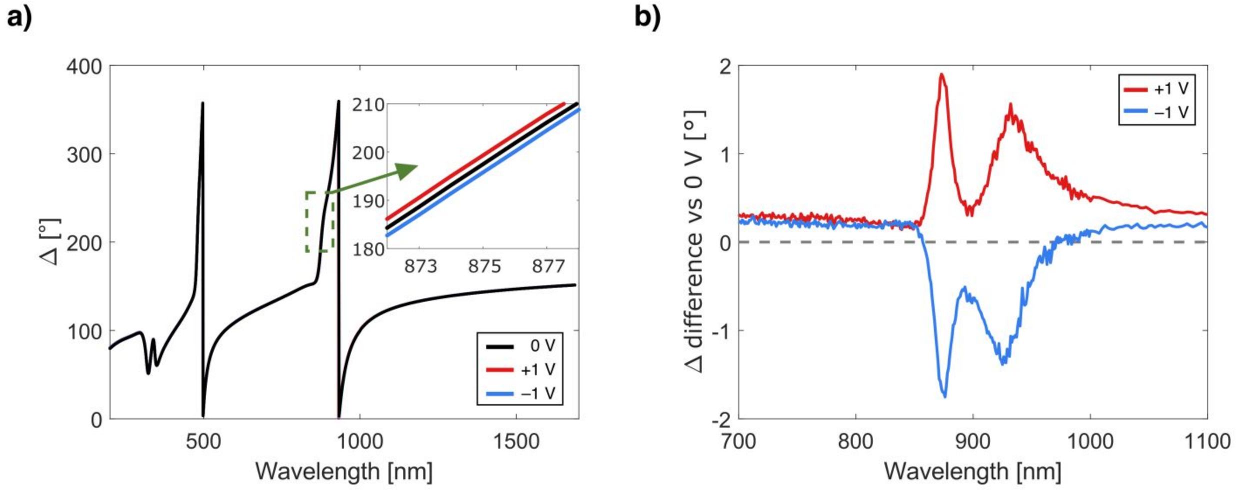

Fig. S1. (a) The ellipsometric parameter Δ, measured at an incidence angle of 65°, obtained under different bias conditions. The inset provides a close-up view of one of the optical resonances. (b) Spectral variation in the Δ function for positive and negative bias voltages.

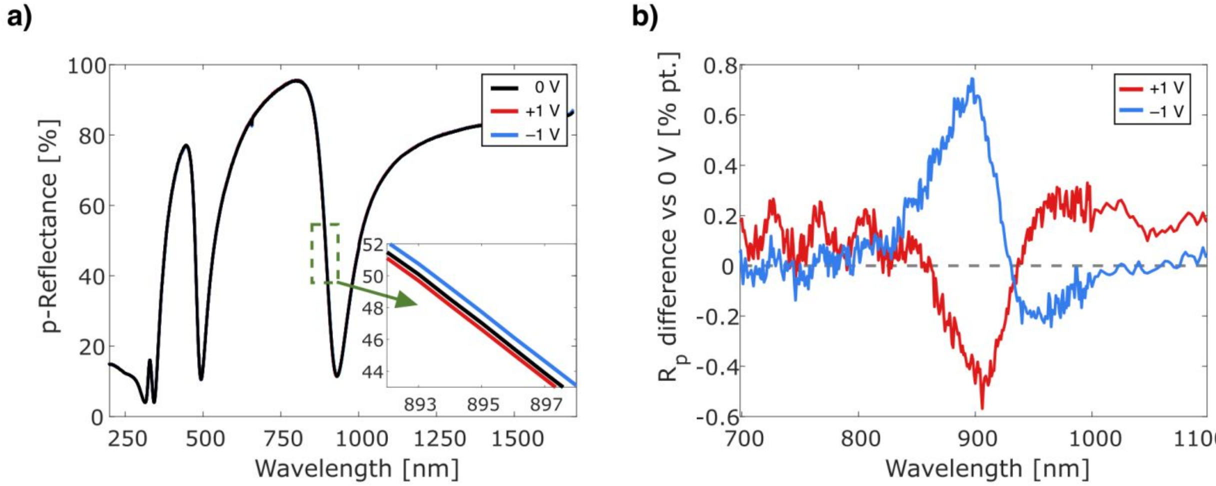

Fig. S2. (a) p-Reflectance, measured at an incidence angle of 65°, obtained under different bias conditions. The inset provides a close-up view of one of the optical resonances. (b) Spectral variation in the p-reflectance for positive and negative bias voltages.

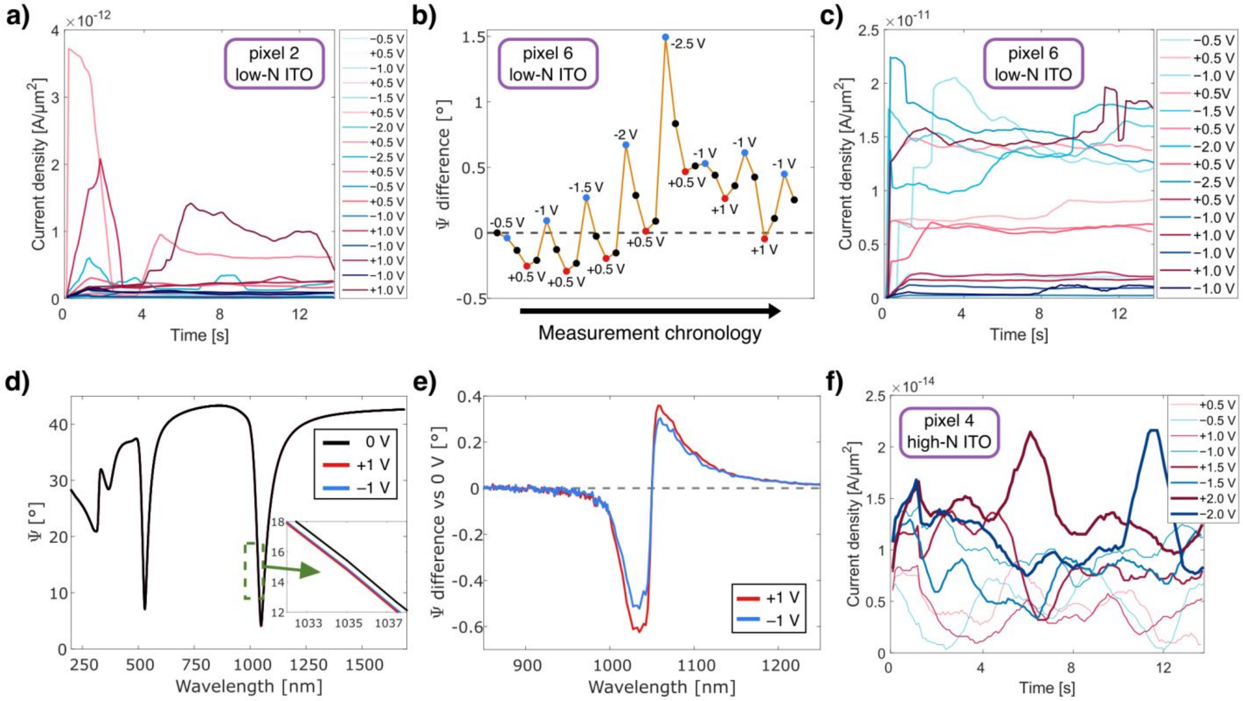

Fig. S3. a) Steady-state current response under different voltage levels for pixel #2, corresponding to the Fig. 2c in the main article. b) Reproducibility of the asymmetrical voltage modulation pattern (similar to pixel #2 as depicted in Fig. 2c in the main article) of Ψ function under applied voltage for pixel #6 at λ =

890 nm and c) the corresponding current flow through the structure. d) The ellipsometric parameter Ψ over wavelength for the pixel #4 with high electron density in ITO layer. Measured at an incidence angle of 65°. e) Spectral variation in the Ψ for positive and negative bias voltages for pixel #4. f) Current flowing through the structure under applied voltage, corresponding to the Fig.2f in the main article.

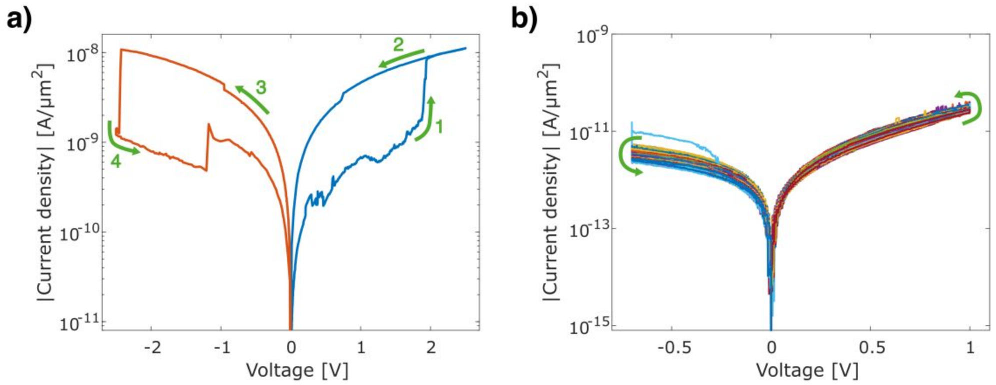

Fig. S4. Current–voltage characteristics of (a) pixel 3, clearly showing typical memristive behavior with distinct high- and low-resistance states, and (b) pixel 5 under multiple modulation cycles (below the resistance switching threshold).

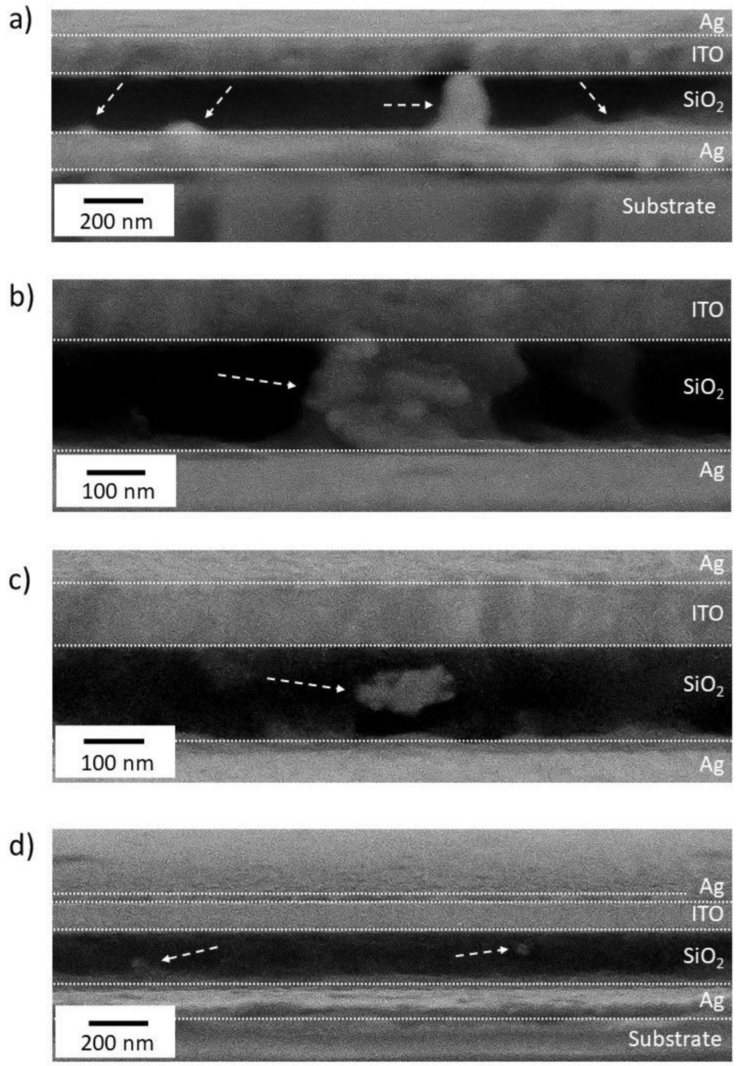

Fig. S5. a) SEM images showing different stages of filament formation: metallic filaments in the SiO_2 layer of pixel #3 (a, b) and silver nanoclusters representing early dendrite growth in pixel #1 (c, d), both recorded after several electrical cycles. White arrows indicate filaments or nanoclusters, while horizontal white lines mark approximate region boundaries. The top electrode appears thicker than in reality because of the slightly inclined imaging geometry.

Additional Discussion

Electric write – optical readout. In our device, information is electrically encoded through two complementary mechanisms: volatile carrier accumulation/depletion (CAL/CDL) and nonvolatile electrochemical metallization (ECM). At the same time, an optical readout, implemented in parallel with electrical excitation, offers a reliable method for monitoring the state of the device. This operation mode—electric write combined with optical readout—offers several key advantages. It enables analog multilevel operation required for neuromorphic and in-memory computing, mitigates the impact of stochastic filament dynamics, and provides a non-invasive, contactless readout that minimizes device stress. Multilevel operation arises from the ability of optical detection to resolve intermediate states, even when the electrical characteristics remain confined to HRS or LRS. The robustness of optical readout originates from its fundamentally different sensing principle. While electrical measurements are strongly affected by the stochastic nature of filament nucleation and rupture, the optical probe integrates over the entire active region, responding to both carrier redistribution and filament-induced modifications of the dielectric environment. Consequently, even if local conduction paths evolve randomly, the optical signal reflects the averaged structural and electronic changes and therefore remains stable and reproducible.

Bridging Short- and Long-Term Plasticity. The coexistence of volatile (CAL/CDL-based) and nonvolatile (ECM-based) states within a single device offers a unique and highly advantageous functionality. The volatile states, governed by fast electronic carrier accumulation and depletion, provide rapid and reversible conductance modulation that naturally emulates short-term synaptic plasticity (STSP). Such behavior is especially valuable for tasks requiring fast, stimulus-dependent adaptation, including real-time signal filtering, event-driven sensory preprocessing, short-term memory buffering, and reservoir-type computing, where transient dynamics enrich the computational capacity of the system without committing changes to long-term memory. Because these responses are electronic rather than ionic in origin, they are significantly faster than ECM-type processes and can operate efficiently at very low currents, thereby reducing power consumption during high-speed operation. In contrast, the nonvolatile states originate from the formation and stabilization of conductive filaments through the ECM mechanism, providing robust, persistent, and multilevel long-term memory (LTSP). These states are essential for weight storage and analog or discretized weight programming in neural networks, supporting stable learning, retention, and inference in hardware implementations of deep neural networks (DNNs) and spiking neural networks (SNNs). The ability to incrementally lower or raise the nonvolatile state by applying negative or positive bias pulses, respectively, enables precise and energy-efficient multilevel tuning of synaptic weights. This makes the device well-suited for vector–matrix multiplication in memristive crossbar arrays, logic-in-memory architectures, and long-term knowledge storage. Importantly, the combination of volatile and nonvolatile behaviors within a

single element bridges short- and long-term plasticity in hardware, reducing the need for complex co-integration of separate device types. While the volatile branch ensures adaptability and dynamic responsiveness, the nonvolatile branch provides stability and retention. Moreover, the use of a robust optical readout parallel to electrical excitation allows intermediate and multilevel states to be resolved with high fidelity, while remaining insensitive to the stochasticity of dendrite formation and rupture. Together, this dual-mode operation supports multifunctional neuromorphic computing: adaptive, event-driven processing and transient dynamics from CAL/CDL, coupled with stable, long-term memory storage from ECM. This hybrid functionality closely mirrors the coexistence of short-term and long-term plasticity in biological synapses, offering a promising route toward energy-efficient, brain-inspired computational hardware.

ITO as an Ionic Barrier. When an ITO layer is introduced between the top electrode and the SiO_2, the ion transport process is modified in two principal ways. First, although silver can still oxidize at the Ag/ITO boundary, the dense oxide lattice of ITO strongly restricts the transport of Ag^+ ions compared to SiO_2. Second, because the potential drop across ITO is smaller than that across SiO_2, the resulting electric field within ITO is weaker, which lowers ion mobility in the bulk of ITO and thereby reduces the driving force for Ag^+ injection from the top electrode into SiO_2. The impact of this mechanism is expected to increase with rising carrier concentration in ITO. Notably, even our "low-N ITO" exhibits carrier concentrations equal to or higher than those typically used in photovoltaic applications, suggesting that even in this case the ITO layer effectively behaves as an active electrode, with the internal electric field strongly suppressed. This could explain why the ionic effects show no significant differences between our low-N and high-N samples.

Taken together, the restricted Ag^+ transport across ITO and the suppression of the internal electric field both limit ion penetration into SiO_2, thereby slowing or even preventing filament nucleation under negative bias. Under positive bias applied to the bottom electrode, Ag^+ ions can be generated without hindrance at the Ag/SiO_2 interface, since SiO_2 is directly in contact with the bottom electrode. However, because the ITO layer is conductive and, as noted above, acts as a barrier to ion transport, ion reduction is not expected at the Ag/ITO interface. Instead, it likely occurs within the SiO_2 layer, between the ITO/SiO_2 and Ag/SiO_2 interfaces, depending on the dominant growth mode.

Impact of CAL on ECM.

It is worth considering whether the presence of the interfacial carrier accumulation layer (CAL) may influence ionic transport in the Ag/ITO/SiO_2/Ag structure. This influence is likely indirect and more difficult to capture than effects associated with classical bulk diffusion. Although the CAL strongly

modifies the local electric field and electrochemical potential at the ITO/SiO_2 interface, SEM images clearly indicate that the dominant ionic transport in the device occurs only from the bottom electrode towards the ITO/SiO_2 interface, and not further in the direction of the top electrode. Since increased carrier concentration in the bulk ITO layer does not induce significant changes in ion transport within the volume, we do not expect the CAL, interfacial effect to play a significant role in ionic transport either. On the other hand, fully developed dendrites resulting from the ECM effect lead to short-circuiting of the system and the gradual disappearance of the CAL/CDL effect.

Growth model of the filaments. The fact that dendrites in our structure seem to initiate growth from the Ag/ITO interface may indicate a bootstrapping growth mode. In this model, Ag nanoclusters formed inside the dielectric act as bipolar nanoelectrodes: they undergo oxidation on their upstream side and reduction on their downstream side, effectively "handing over" material to newly formed clusters located closer to the counter electrode. As a result, filament growth proceeds in a step-by-step manner rather than through continuous reduction at the ITO/SiO_2 interface. This interpretation further suggests that the direct reduction of Ag^+ at the ITO/SiO_2 boundary may be less efficient than at inert metallic electrodes, thereby favoring a cluster-mediated, bootstrapped growth pathway.

Conditions of controllable switching. At low applied voltages ($\leq \pm 0.5$–1 V), the response is dominated by fast, volatile carrier accumulation/depletion (CAL/CDL) at the ITO/SiO_2 interface. Electrochemical metallization processes are also initiated, involving Ag^+ ion migration within the SiO_2 layer, but without the formation of complete dendrites. As a result, the corresponding state changes can be detected optically but not electrically. In this regime, the modulation is fully reversible, with the optical signal revealing both volatile and nonvolatile components of operation.

At higher voltages ($\geq \pm 2$ V), ECM processes become strongly manifested, with full dendrite formation taking place. This leads to nonvolatile, persistent changes in the optical response, accompanied by the characteristic hysteretic I–V behavior of memristive devices, but also to the suppression of volatile CAL/CDL effects due to short-circuiting of the device. In this regime, the states can be monitored both electrically and optically; however, voltages exceeding ~2 V may result in excessive dendrite stabilization and hinder the return to the electrical HRS state.

Methods

Sample fabrication. The device was fabricated entirely using an electron beam PVD system (Lesker 75). The process began with the preparation of a commercial glass substrate (Ossila ultra-flat quartz-coated glass), which was cleaned using isopropanol, deionized water, and oxygen plasma to remove any residual

dust particles. Subsequently, a 100 nm bottom silver electrode, a 180 nm SiO_2 layer, 100 nm ITO layer and a 20 nm top silver electrode were deposited sequentially. Ag and SiO_2 deposition processes were conducted under standard evaporation conditions at room temperature (22 °C) and a 5 x 10^{-5} Torr pressure and evaporation speed 1 Å/s and 2 Å/s respectively. ITO thin films were deposited onto quartz substrates heated to 80 °C. The deposition rate was fixed at 10 Å/s. Importantly, this method does not require any post-annealing, which makes it unique compared to conventional ITO fabrication routes. Two series of samples were prepared by varying the oxygen plasma conditions:

- Low-carrier-concentration ITO: oxygen flow = 5 sccm, discharge voltage = 150 V, discharge current = 0.4 A.
- High-carrier-concentration ITO: oxygen flow = 3 sccm, discharge voltage = 150 V, discharge current = 0.3 A.

This controlled adjustment of plasma parameters enabled reproducible tuning of the carrier concentration from low to high values, while maintaining smooth morphology.
To arrange the layers, we employed 3D-printed shadow masks, which were swapped after each layer to achieve the desired geometry. The mask-changing process was performed in a laminar flow chamber to prevent any contaminants from settling on the surface of the layers. Each sample consisted of six pixels (areas where all layers overlap, approximately 2.5 x 2.5 mm), with separate electrical contacts (pure indium) soldered to the bottom electrode of each pixel. The top electrode was common to all pixels, with only one electrical contact soldered to this layer.

Ellipsometric measurements and modeling. The optical properties of the devices were investigated using an RC2 ellipsometer (J.A. Woollam Co.) equipped with dual-rotating compensators located before and after the sample. Measurements were performed with focusing probes, reducing the beam spot size on the sample surface to ~400 μm. A typical measurement protocol for each pixel was as follows. First, prior to voltage application, Ψ and Δ spectra were recorded at three angles of incidence (60°, 65°, and 70°), serving as the baseline for constructing an optical model of the structure. During electrical pulse stimulation, however, measurements were restricted to 65°, since the ellipsometric acquisition time was 13 s and we aimed to minimize the risk of state changes occurring between measurements at different angles. Each Ψ and Δ acquisition was accompanied by a simultaneous intensity reflectance measurement.

To describe the data, a layered optical model of the structure was developed. Tests showed that a simplified six-layer stack—comprising the top electrode, bulk semiconductor, a carrier accumulation/depletion layer within the semiconductor, dielectric, bottom electrode, and substrate—with a roughness layer on top was sufficient to capture the material's response. More complex models, including graded layers, mixed

compositions, or additional sublayers, did not provide a significant improvement in terms of mean squared error (MSE). The dielectric permittivity functions were modeled using a general oscillator approach for the electrodes (three harmonic oscillators plus a Drude term), a Tauc–Lorentz + Drude model for the ITO, and a three-term Sellmeier formula for SiO_2. The accuracy of this approach was validated by the resulting MSE values, which were on the order of ~5.

Electrical characterization. For the I-V measurements and steady-state current response under different voltage levels, a voltage was applied to the selected pixel using a Rohde & Schwarz HMP 2020 power supply with a precision of 0.001 V. The current was measured with a Keithley Digital Multimeter 2701 with a resolution of 10 nA, and the compliance current was set to 200 mA. Each data point was acquired over approximately 0.02 seconds (1 PLC).
